# 4-(4-Chloro­phen­yl)-4-hy­droxy­piperidinium benzoate

**DOI:** 10.1107/S1600536811017855

**Published:** 2011-05-20

**Authors:** Jerry P. Jasinski, James A. Golen, B. P. Siddaraju, A. S. Dayananda, H. S. Yathirajan

**Affiliations:** a Department of Chemistry, Keene State College, 229 Main Street, Keene, NH 03435-2001, USA; b Department of Studies in Chemistry, University of Mysore, Manasagangotri, Mysore 570 006, India

## Abstract

In the title salt, C_11_H_15_ClNO^+^·C_7_H_5_O_2_^−^, the dihedral angle between the mean planes of the chloro­phenyl ring of the cation and the benzene ring of the anion is 74.4 (1)°. In the cation, the six-membered piperazine ring adopts a chair conformation. The crystal packing is stabilized by inter­molecular N—H⋯O and O—H⋯O hydrogen bonds, and weak inter­molecular C—H⋯O, C—H⋯Cl and C—H⋯π inter­actions.

## Related literature

For the synthesis and biological activity of uncondensed cyclic derivatives of piperidine, see: Vartanyan (1984 ▶). For puckering parameters, see: Cremer & Pople (1975 ▶) For related structures, see: Jasinski *et al.* (2009 ▶). For ring-motif pattterns, see: Bernstein *et al.* (1994 ▶). 
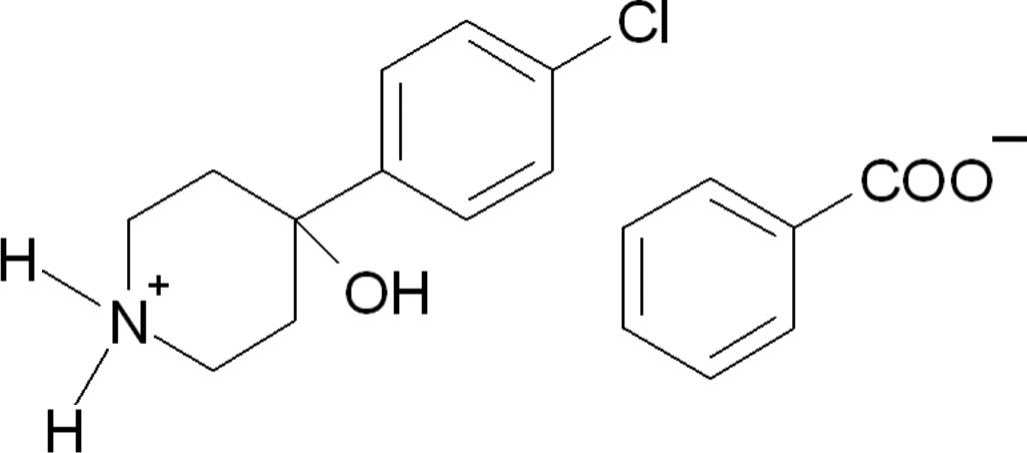


## Experimental

### 

#### Crystal data

C_11_H_15_ClNO^+^·C_7_H_5_O_2_^−^
*M* *_r_* = 333.80
Triclinic, 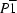

*a* = 9.6235 (12) Å
*b* = 10.0971 (16) Å
*c* = 10.2251 (14) Å
α = 99.608 (12)°
β = 108.748 (13)°
γ = 113.357 (14)°
*V* = 812.7 (2) Å^3^
*Z* = 2
Mo *K*α radiation
μ = 0.25 mm^−1^
*T* = 173 K
0.34 × 0.30 × 0.13 mm

#### Data collection

Oxford Diffraction Xcalibur Eos Gemini diffractometer
Absorption correction: multi-scan (*CrysAlis RED*; Oxford Diffraction, 2010 ▶) *T*_min_ = 0.920, *T*_max_ = 0.968
7857 measured reflections
4184 independent reflections
3222 reflections with *I* > 2σ(*I*)
*R*_int_ = 0.018

#### Refinement

*R*[*F* ^2^ > 2σ(*F* ^2^)] = 0.046
*wR*(*F* ^2^) = 0.125
*S* = 1.05
4184 reflections
217 parameters
4 restraints
H atoms treated by a mixture of independent and constrained refinement
Δρ_max_ = 0.29 e Å^−3^
Δρ_min_ = −0.34 e Å^−3^

### 

Data collection: *CrysAlis PRO* (Oxford Diffraction, 2010 ▶); cell refinement: *CrysAlis PRO*; data reduction: *CrysAlis RED* (Oxford Diffraction, 2010 ▶); program(s) used to solve structure: *SHELXS97* (Sheldrick, 2008 ▶); program(s) used to refine structure: *SHELXL97* (Sheldrick, 2008 ▶); molecular graphics: *SHELXTL* (Sheldrick, 2008 ▶); software used to prepare material for publication: *SHELXTL*.

## Supplementary Material

Crystal structure: contains datablocks global, I. DOI: 10.1107/S1600536811017855/pv2415sup1.cif

Structure factors: contains datablocks I. DOI: 10.1107/S1600536811017855/pv2415Isup2.hkl

Supplementary material file. DOI: 10.1107/S1600536811017855/pv2415Isup3.cml

Additional supplementary materials:  crystallographic information; 3D view; checkCIF report

## Figures and Tables

**Table 1:** Hydrogen-bond geometry (Å, °) *Cg*3 is the centroid of the C13–C18 ring.

*D*—H⋯*A*	*D*—H	H⋯*A*	*D*⋯*A*	*D*—H⋯*A*
N1—H1*NA*⋯O3^i^	0.87 (1)	1.84 (1)	2.6964 (17)	166 (2)
O1—H1*O*⋯O3	0.82 (2)	2.05 (2)	2.7780 (16)	147 (2)
N1—H1*NB*⋯O2^ii^	0.86 (1)	1.92 (1)	2.7609 (18)	166 (2)
C16—H16*A*⋯Cl1^iii^	0.95	2.78	3.5268 (17)	136
C9—H9*B*⋯O1^i^	0.99	2.46	3.3008 (19)	143
C1—H1*A*⋯*Cg*3^iv^	0.95	2.70	3.554 (2)	150

Symmetry codes: (i) 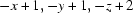; (ii) 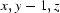; (iii) 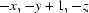; (iv) 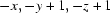.

**Fig. 1.**
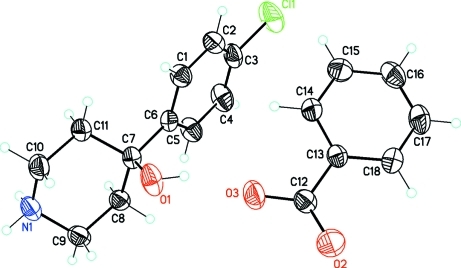
Molecular structure of the title compound showing the atom labeling scheme and 50% probability displacement ellipsoids.

**Fig. 2.**
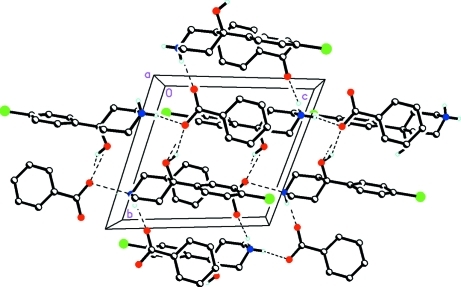
Packing diagram for the title compound viewed down the a axis. Dashed lines indicate N—H···O and O—H···O hydrogen bonds generating one dimensional chains along the c axis.

## supplementary crystallographic information

### Comment

4-(4-Chlorophenyl)-4-hydroxypiperidine is used as an intermediate for the synthesis of pharmaceuticals such as haloperidol (a neuroleptic drug used to treat patients with psychotic illnesses, extreme agitation, or Tourette's syndrome) and loperamide (a synthetic piperidine derivative, effective against diarrhea resulting from gastroenteritis or inflammatory bowel disease). A review on the synthesis and biological activities of uncondensed cyclic derivatives of piperidine is reported (Vartanyan, 1984).  The crystal structure of a related compound, 4-[(E)-(2,4-difluorophenyl) (hydroxyimino)methyl]piperidinium picrate (Jasinski *et al.*, 2009) has been reported. In this paperwe report the crystal structure of C_11_H_15_ONCl^+^ . C_15_H_12_O_2_^-^.

In the title salt (Fig. 1), the 6-membered piperazine ring in the cation adopts a chair conformation with puckering parameters (Cremer & Pople, 1975) *Q*, θ and φ, 0.568 (2) Å, 0.00 (19)° and 278 (9)°, respectively . The dihedral angle between the mean planes of the chlorophenyl ring of the cation and the benzene ring of the anion is 74.4 (1)°. The crystal structure is stabilized by N1—H1NA···O3 and N1—H1NB···O2, hydrogen bonds forming R_4_^4^(12) ring-motif patttern (Bernstein *et al.*, 1994) and N1—H1NA···O3, O1—H10···O3 hydrogen bonds resulting in R_2_^4^(16) ring-motif, generating one dimensional chains along the *c* axis (Fig. 2). The structure is further consolidated by weak C9—H9B···O1, C16—H16A···Cl1 and C1—H1A···Cg3 π-ring intermolecular interactions.

### Experimental

Solutions of 4-(4-chlorophenyl)-piperidin-4-ol (2.12 g, 0.01 mol) in methanol (10 ml) and benzoic acid (1.226 g, 0.01 mol) in methanol (10 ml) were mixed and stirred in a beaker at 333 K for 30 minutes.  The mixture was kept aside for three days at room temperature. The salt thus obtained was filtered and dried in a vaccum desiccator over phosphorous pentoxide. The compound was recrystallized from N,N-dimethylformamide by slow evaporation (m.p: 498 - 501 K).

### Refinement

Hydrogen atoms on O1 and N1 were found from a Fourier difference map and were refined using DFIX 0.84(0.02) and 0.86(0.01) values for O–H and N–H distances, respectively, and *U*_iso_(H) = 1.2 times *U*_eq_ (O/N). The rest of the H atoms were positioned geometrically, and allowed to ride on their parent atoms, with C—H distances 0.95 Å (CH) or 0.99 Å (CH_2_) and *U*_iso_(H) = 1.18-1.21 times *U*_eq_ (C).

### Crystal data

**Table:**

C_11_H_15_ClNO^+^·C_7_H_5_O_2_^−^	*Z* = 2
*M**_r_* = 333.80	*F*(000) = 352
Triclinic, *P*1	*D*_x_ = 1.364 Mg m^−^^3^
Hall symbol: -P 1	Mo *K*α radiation, λ = 0.71073 Å
*a* = 9.6235 (12) Å	Cell parameters from 3721 reflections
*b* = 10.0971 (16) Å	θ = 3.7–32.3°
*c* = 10.2251 (14) Å	µ = 0.25 mm^−^^1^
α = 99.608 (12)°	*T* = 173 K
β = 108.748 (13)°	Block, colorless
γ = 113.357 (14)°	0.34 × 0.30 × 0.13 mm
*V* = 812.7 (2) Å^3^	

### Data collection

**Table:**

Oxford Diffraction Xcalibur Eos Gemini diffractometer	4184 independent reflections
Radiation source: Enhance (Mo) X-ray Source	3222 reflections with *I* > 2σ(*I*)
graphite	*R*_int_ = 0.018
Detector resolution: 16.1500 pixels mm^-1^	θ_max_ = 28.7°, θ_min_ = 3.7°
ω scans	*h* = −12→12
Absorption correction: multi-scan (*CrysAlis RED*; Oxford Diffraction, 2010)	*k* = −13→13
*T*_min_ = 0.920, *T*_max_ = 0.968	*l* = −13→13
7857 measured reflections	

### Refinement

**Table:**

Refinement on *F*^2^	Primary atom site location: structure-invariant direct methods
Least-squares matrix: full	Secondary atom site location: difference Fourier map
*R*[*F*^2^ > 2σ(*F*^2^)] = 0.046	Hydrogen site location: inferred from neighbouring sites
*wR*(*F*^2^) = 0.125	H atoms treated by a mixture of independent and constrained refinement
*S* = 1.05	*w* = 1/[σ^2^(*F*_o_^2^) + (0.0558*P*)^2^ + 0.1776*P*] where *P* = (*F*_o_^2^ + 2*F*_c_^2^)/3
4184 reflections	(Δ/σ)_max_ < 0.001
217 parameters	Δρ_max_ = 0.29 e Å^−^^3^
4 restraints	Δρ_min_ = −0.34 e Å^−^^3^

### Special details

**Table:**

Geometry. All esds (except the esd in the dihedral angle between two l.s. planes) are estimated using the full covariance matrix. The cell esds are taken into account individually in the estimation of esds in distances, angles and torsion angles; correlations between esds in cell parameters are only used when they are defined by crystal symmetry. An approximate (isotropic) treatment of cell esds is used for estimating esds involving l.s. planes.
Refinement. Refinement of *F*^2^ against ALL reflections. The weighted *R*-factor *wR* and goodness of fit *S* are based on *F*^2^, conventional *R*-factors *R* are based on *F*, with *F* set to zero for negative *F*^2^. The threshold expression of *F*^2^ > σ(*F*^2^) is used only for calculating *R*-factors(gt) *etc*. and is not relevant to the choice of reflections for refinement. *R*-factors based on *F*^2^ are statistically about twice as large as those based on *F*, and *R*- factors based on ALL data will be even larger.

### Fractional atomic coordinates and isotropic or equivalent isotropic displacement parameters (Å^2^)

**Table:**

	*x*	*y*	*z*	*U*_iso_*/*U*_eq_	
Cl1	0.01350 (8)	0.19249 (7)	0.06335 (5)	0.06605 (19)	
O1	0.37436 (18)	0.44147 (14)	0.79300 (12)	0.0470 (3)	
H1O	0.360 (3)	0.498 (2)	0.746 (2)	0.056*	
O2	0.46446 (18)	0.94763 (13)	0.77613 (13)	0.0553 (4)	
O3	0.36182 (17)	0.70222 (13)	0.75160 (12)	0.0504 (3)	
N1	0.51659 (18)	0.21683 (15)	0.95370 (13)	0.0372 (3)	
H1NB	0.514 (2)	0.1342 (16)	0.9106 (18)	0.045*	
H1NA	0.561 (2)	0.231 (2)	1.0473 (12)	0.045*	
C1	0.1141 (2)	0.24120 (18)	0.48209 (16)	0.0351 (3)	
H1A	0.0551	0.2344	0.5416	0.042*	
C2	0.0314 (2)	0.21412 (19)	0.33410 (17)	0.0393 (4)	
H2A	−0.0832	0.1888	0.2920	0.047*	
C3	0.1181 (2)	0.22451 (18)	0.24891 (16)	0.0387 (4)	
C4	0.2839 (2)	0.2611 (2)	0.30731 (18)	0.0474 (4)	
H4A	0.3419	0.2678	0.2470	0.057*	
C5	0.3653 (2)	0.2881 (2)	0.45636 (17)	0.0404 (4)	
H5A	0.4802	0.3140	0.4978	0.048*	
C6	0.28210 (18)	0.27817 (15)	0.54568 (14)	0.0278 (3)	
C7	0.36689 (18)	0.30769 (15)	0.70961 (14)	0.0282 (3)	
C8	0.54504 (19)	0.32943 (17)	0.76015 (15)	0.0331 (3)	
H8A	0.5421	0.2395	0.7006	0.040*	
H8B	0.6156	0.4212	0.7432	0.040*	
C9	0.6232 (2)	0.34823 (18)	0.92121 (16)	0.0373 (3)	
H9A	0.7351	0.3556	0.9474	0.045*	
H9B	0.6383	0.4445	0.9818	0.045*	
C10	0.3447 (2)	0.19647 (19)	0.91069 (16)	0.0388 (4)	
H10A	0.3496	0.2880	0.9697	0.047*	
H10B	0.2763	0.1064	0.9310	0.047*	
C11	0.26339 (19)	0.17363 (18)	0.74911 (16)	0.0346 (3)	
H11A	0.1503	0.1632	0.7235	0.042*	
H11B	0.2503	0.0774	0.6904	0.042*	
C12	0.37590 (19)	0.80987 (17)	0.70139 (15)	0.0324 (3)	
C13	0.27602 (18)	0.76646 (16)	0.53903 (15)	0.0283 (3)	
C14	0.1622 (2)	0.61404 (17)	0.45411 (16)	0.0363 (3)	
H14A	0.1524	0.5367	0.4977	0.044*	
C15	0.0633 (2)	0.5739 (2)	0.30681 (18)	0.0447 (4)	
H15A	−0.0151	0.4695	0.2498	0.054*	
C16	0.0782 (2)	0.6857 (2)	0.24240 (18)	0.0463 (4)	
H16A	0.0093	0.6580	0.1413	0.056*	
C17	0.1924 (2)	0.8366 (2)	0.32407 (19)	0.0452 (4)	
H17A	0.2042	0.9130	0.2790	0.054*	
C18	0.2902 (2)	0.87754 (17)	0.47237 (17)	0.0360 (3)	
H18A	0.3677	0.9823	0.5289	0.043*	

### Atomic displacement parameters (Å^2^)

**Table:**

	*U*^11^	*U*^22^	*U*^33^	*U*^12^	*U*^13^	*U*^23^
Cl1	0.0874 (4)	0.0854 (4)	0.0294 (2)	0.0485 (3)	0.0179 (2)	0.0257 (2)
O1	0.0826 (9)	0.0447 (7)	0.0316 (6)	0.0468 (7)	0.0235 (6)	0.0149 (5)
O2	0.0710 (9)	0.0364 (6)	0.0391 (6)	0.0248 (6)	0.0082 (6)	0.0014 (5)
O3	0.0727 (9)	0.0397 (6)	0.0299 (5)	0.0272 (6)	0.0101 (6)	0.0147 (5)
N1	0.0512 (8)	0.0349 (7)	0.0253 (6)	0.0256 (6)	0.0101 (6)	0.0098 (5)
C1	0.0360 (8)	0.0427 (8)	0.0311 (7)	0.0198 (7)	0.0169 (6)	0.0158 (6)
C2	0.0362 (8)	0.0442 (9)	0.0344 (7)	0.0189 (7)	0.0109 (7)	0.0159 (7)
C3	0.0526 (10)	0.0401 (8)	0.0270 (7)	0.0256 (8)	0.0151 (7)	0.0155 (6)
C4	0.0594 (11)	0.0691 (12)	0.0375 (8)	0.0397 (10)	0.0314 (8)	0.0291 (8)
C5	0.0406 (9)	0.0589 (10)	0.0371 (8)	0.0300 (8)	0.0225 (7)	0.0243 (7)
C6	0.0346 (8)	0.0273 (6)	0.0269 (6)	0.0175 (6)	0.0146 (6)	0.0119 (5)
C7	0.0360 (8)	0.0289 (7)	0.0260 (6)	0.0192 (6)	0.0153 (6)	0.0109 (5)
C8	0.0326 (8)	0.0347 (7)	0.0294 (7)	0.0152 (6)	0.0119 (6)	0.0102 (6)
C9	0.0364 (8)	0.0387 (8)	0.0287 (7)	0.0172 (7)	0.0075 (6)	0.0080 (6)
C10	0.0466 (9)	0.0422 (8)	0.0329 (7)	0.0217 (7)	0.0192 (7)	0.0192 (6)
C11	0.0342 (8)	0.0379 (8)	0.0320 (7)	0.0159 (7)	0.0142 (6)	0.0164 (6)
C12	0.0366 (8)	0.0331 (7)	0.0290 (7)	0.0210 (6)	0.0116 (6)	0.0083 (6)
C13	0.0298 (7)	0.0322 (7)	0.0300 (7)	0.0187 (6)	0.0150 (6)	0.0129 (5)
C14	0.0387 (8)	0.0330 (7)	0.0326 (7)	0.0155 (7)	0.0110 (6)	0.0139 (6)
C15	0.0445 (10)	0.0408 (9)	0.0335 (8)	0.0149 (8)	0.0079 (7)	0.0089 (7)
C16	0.0498 (10)	0.0602 (11)	0.0316 (8)	0.0290 (9)	0.0141 (7)	0.0216 (8)
C17	0.0557 (11)	0.0527 (10)	0.0442 (9)	0.0311 (9)	0.0271 (8)	0.0316 (8)
C18	0.0406 (9)	0.0327 (7)	0.0407 (8)	0.0191 (7)	0.0205 (7)	0.0166 (6)

### Geometric parameters (Å, °)

**Table:**

Cl1—C3	1.7400 (15)	C8—C9	1.5167 (19)
O1—C7	1.4338 (17)	C8—H8A	0.9900
O1—H1O	0.823 (15)	C8—H8B	0.9900
O2—C12	1.2376 (19)	C9—H9A	0.9900
O3—C12	1.2560 (18)	C9—H9B	0.9900
N1—C10	1.485 (2)	C10—C11	1.514 (2)
N1—C9	1.487 (2)	C10—H10A	0.9900
N1—H1NB	0.861 (10)	C10—H10B	0.9900
N1—H1NA	0.873 (10)	C11—H11A	0.9900
C1—C2	1.383 (2)	C11—H11B	0.9900
C1—C6	1.393 (2)	C12—C13	1.5064 (19)
C1—H1A	0.9500	C13—C14	1.389 (2)
C2—C3	1.376 (2)	C13—C18	1.391 (2)
C2—H2A	0.9500	C14—C15	1.381 (2)
C3—C4	1.373 (3)	C14—H14A	0.9500
C4—C5	1.391 (2)	C15—C16	1.381 (2)
C4—H4A	0.9500	C15—H15A	0.9500
C5—C6	1.387 (2)	C16—C17	1.373 (3)
C5—H5A	0.9500	C16—H16A	0.9500
C6—C7	1.5250 (18)	C17—C18	1.386 (2)
C7—C8	1.533 (2)	C17—H17A	0.9500
C7—C11	1.536 (2)	C18—H18A	0.9500
			
C7—O1—H1O	114.0 (15)	N1—C9—H9A	109.4
C10—N1—C9	111.70 (12)	C8—C9—H9A	109.4
C10—N1—H1NB	110.7 (13)	N1—C9—H9B	109.4
C9—N1—H1NB	110.2 (13)	C8—C9—H9B	109.4
C10—N1—H1NA	108.5 (13)	H9A—C9—H9B	108.0
C9—N1—H1NA	110.3 (13)	N1—C10—C11	110.57 (13)
H1NB—N1—H1NA	105.3 (16)	N1—C10—H10A	109.5
C2—C1—C6	121.38 (14)	C11—C10—H10A	109.5
C2—C1—H1A	119.3	N1—C10—H10B	109.5
C6—C1—H1A	119.3	C11—C10—H10B	109.5
C3—C2—C1	118.85 (15)	H10A—C10—H10B	108.1
C3—C2—H2A	120.6	C10—C11—C7	112.01 (13)
C1—C2—H2A	120.6	C10—C11—H11A	109.2
C4—C3—C2	121.62 (14)	C7—C11—H11A	109.2
C4—C3—Cl1	119.92 (13)	C10—C11—H11B	109.2
C2—C3—Cl1	118.46 (13)	C7—C11—H11B	109.2
C3—C4—C5	118.82 (15)	H11A—C11—H11B	107.9
C3—C4—H4A	120.6	O2—C12—O3	124.52 (14)
C5—C4—H4A	120.6	O2—C12—C13	118.42 (13)
C6—C5—C4	121.26 (15)	O3—C12—C13	117.06 (13)
C6—C5—H5A	119.4	C14—C13—C18	118.72 (13)
C4—C5—H5A	119.4	C14—C13—C12	120.11 (13)
C5—C6—C1	118.06 (13)	C18—C13—C12	121.12 (13)
C5—C6—C7	122.92 (13)	C15—C14—C13	120.57 (14)
C1—C6—C7	119.01 (12)	C15—C14—H14A	119.7
O1—C7—C6	110.41 (11)	C13—C14—H14A	119.7
O1—C7—C8	108.40 (12)	C16—C15—C14	120.02 (16)
C6—C7—C8	112.93 (11)	C16—C15—H15A	120.0
O1—C7—C11	106.31 (12)	C14—C15—H15A	120.0
C6—C7—C11	110.12 (12)	C17—C16—C15	120.19 (15)
C8—C7—C11	108.43 (11)	C17—C16—H16A	119.9
C9—C8—C7	112.22 (12)	C15—C16—H16A	119.9
C9—C8—H8A	109.2	C16—C17—C18	119.94 (14)
C7—C8—H8A	109.2	C16—C17—H17A	120.0
C9—C8—H8B	109.2	C18—C17—H17A	120.0
C7—C8—H8B	109.2	C17—C18—C13	120.54 (15)
H8A—C8—H8B	107.9	C17—C18—H18A	119.7
N1—C9—C8	111.13 (12)	C13—C18—H18A	119.7
			
C6—C1—C2—C3	−0.1 (2)	C10—N1—C9—C8	−56.75 (16)
C1—C2—C3—C4	−0.1 (2)	C7—C8—C9—N1	55.69 (16)
C1—C2—C3—Cl1	−179.23 (12)	C9—N1—C10—C11	57.50 (16)
C2—C3—C4—C5	0.0 (3)	N1—C10—C11—C7	−57.35 (17)
Cl1—C3—C4—C5	179.15 (13)	O1—C7—C11—C10	−61.50 (15)
C3—C4—C5—C6	0.3 (3)	C6—C7—C11—C10	178.89 (12)
C4—C5—C6—C1	−0.5 (2)	C8—C7—C11—C10	54.87 (16)
C4—C5—C6—C7	−179.69 (14)	O2—C12—C13—C14	−173.36 (15)
C2—C1—C6—C5	0.4 (2)	O3—C12—C13—C14	6.1 (2)
C2—C1—C6—C7	179.65 (13)	O2—C12—C13—C18	3.9 (2)
C5—C6—C7—O1	114.43 (16)	O3—C12—C13—C18	−176.65 (15)
C1—C6—C7—O1	−64.80 (17)	C18—C13—C14—C15	−1.0 (2)
C5—C6—C7—C8	−7.11 (19)	C12—C13—C14—C15	176.34 (15)
C1—C6—C7—C8	173.66 (13)	C13—C14—C15—C16	0.7 (3)
C5—C6—C7—C11	−128.48 (15)	C14—C15—C16—C17	0.6 (3)
C1—C6—C7—C11	52.29 (16)	C15—C16—C17—C18	−1.5 (3)
O1—C7—C8—C9	61.09 (15)	C16—C17—C18—C13	1.1 (3)
C6—C7—C8—C9	−176.24 (11)	C14—C13—C18—C17	0.1 (2)
C11—C7—C8—C9	−53.92 (15)	C12—C13—C18—C17	−177.21 (14)

### Hydrogen-bond geometry (Å, °)

**Table:**

Cg3 is the centroid of the C13–C18 ring.

**Table:**

*D*—H···*A*	*D*—H	H···*A*	*D*···*A*	*D*—H···*A*
N1—H1NA···O3^i^	0.87 (1)	1.84 (1)	2.6964 (17)	166 (2)
O1—H1O···O3	0.82 (2)	2.05 (2)	2.7780 (16)	147 (2)
N1—H1NB···O2^ii^	0.86 (1)	1.92 (1)	2.7609 (18)	166 (2)
C16—H16A···Cl1^iii^	0.95	2.78	3.5268 (17)	136
C9—H9B···O1^i^	0.99	2.46	3.3008 (19)	143
C1—H1A···Cg3^iv^	0.95	2.70	3.554 (2)	150

Symmetry codes: (i) −*x*+1, −*y*+1, −*z*+2; (ii) *x*, *y*−1, *z*; (iii) −*x*, −*y*+1, −*z*; (iv) −*x*, −*y*+1, −*z*+1.
